# Hepatitis C Virus Resistance to Carbohydrate-Binding Agents

**DOI:** 10.1371/journal.pone.0149064

**Published:** 2016-02-12

**Authors:** Laure Izquierdo, Catarina Oliveira, Carole Fournier, Véronique Descamps, Virginie Morel, Jean Dubuisson, Etienne Brochot, Catherine Francois, Sandrine Castelain, Gilles Duverlie, Francois Helle

**Affiliations:** 1 EA4294, Laboratoire de Virologie, Centre Universitaire de Recherche en Santé, Centre Hospitalier Universitaire et Université de Picardie Jules Verne, Amiens, France; 2 Univ. Lille, CNRS, Inserm, CHU Lille, Institut Pasteur de Lille, U1019—UMR 8204—CIIL—Centre d'Infection et d'Immunité de Lille, Lille, France; Saint Louis University, UNITED STATES

## Abstract

Carbohydrate binding agents (CBAs), including natural lectins, are more and more considered as broad-spectrum antivirals. These molecules are able to directly inhibit many viruses such as Human Immunodeficiency Virus (HIV), Hepatitis C Virus (HCV), Dengue Virus, Ebola Virus or Severe Acute Respiratory Syndrome Coronavirus through binding to envelope protein N-glycans. In the case of HIV, it has been shown that CBAs select for mutant viruses with N-glycosylation site deletions which are more sensitive to neutralizing antibodies. In this study we aimed at evaluating the HCV resistance to CBAs *in vitro*. HCV was cultivated in the presence of increasing Galanthus nivalis agglutinin (GNA), Cyanovirin-N, Concanavalin-A or Griffithsin concentrations, during more than eight weeks. At the end of lectin exposure, the genome of the isolated strains was sequenced and several potential resistance mutations in the E1E2 envelope glycoproteins were identified. The effect of these mutations on viral fitness as well as on sensitivity to inhibition by lectins, soluble CD81 or the 3/11 neutralizing antibody was assessed. Surprisingly, none of these mutations, alone or in combination, conferred resistance to CBAs. In contrast, we observed that some mutants were more sensitive to 3/11 or CD81-LEL inhibition. Additionally, several mutations were identified in the Core and the non-structural proteins. Thus, our results suggest that in contrast to HIV, HCV resistance to CBAs is not directly conferred by mutations in the envelope protein genes but could occur through an indirect mechanism involving mutations in other viral proteins. Further investigations are needed to completely elucidate the underlying mechanisms.

## Introduction

Hepatitis C virus (HCV) is a small positive single-stranded RNA virus that belongs to the Hepacivirus genus in the *Flaviviridae* family [1]. This enveloped virus infects hepatocytes and causes serious liver diseases in humans. Past treatment of HCV included the use of interferon and ribavirin, a combination that was not very effective and not well tolerated. However, a new era finally started in 2014 thanks to the development of direct-acting antiviral arsenal which enables to achieve a sustained virologic response in more than 90% of treated patients in clinical trials [2]. Nevertheless, some concerns remain such as access to care in low- to middle-income countries or viral resistance that could be encountered in real-life less-compliant populations.

HCV entry into hepatocytes is a complex multistep process that involves viral envelope glycoproteins and several cell entry factors including CD81, SR-BI, CLDN1 and OCLN [3]. E1 and E2 are the two envelope glycoproteins that are present on the surface of viral particles as large covalent complexes stabilized by disulfide bridges [4]. Both glycoproteins are heavily N-glycosylated and, as a result, one third of the molecular mass of E1E2 heterodimers corresponds to N-glycans. Indeed, 4 and 11 N-glycosylation sites are conserved in E1 and E2 sequences from most genotypes and it has been shown that the majority of these sites harbor high-mannose-type glycans, even after egress of viral particles from the cells [4]. In addition, we contributed to demonstrate that the corresponding N-glycans play an important role for the function of these proteins: i) they enable the correct folding of the envelope proteins, ii) they modulate the efficiency of the entry step and iii) they mask conserved neutralizing epitopes on E2 envelope glycoprotein close to the binding site to the cellular receptor CD81 [5–9]. These features make HCV N-glycans promising target for new antiviral strategies, all the more as high-mannose glycans are rarely present on cellular proteins after their exit from the endoplasmic reticulum. A proof of concept has been provided *in vitro* and *in vivo* by using several lectins such as Cyanovirin-N, Griffithsin or Scytovirin as well as the non-peptidic carbohydrate binding agent (CBA) Pradimicin-A [10–15]. However, a potential resistance of HCV to such a therapeutic strategy has never been investigated.

In this study, we sought to evaluate *in vitro* the resistance of HCV to CBAs. To this end, we cultivated HCV JFH1 strain [16] in the presence of increasing concentrations of different lectins (Galanthus nivalis agglutinin [GNA], Cyanovirin-N [CV-N], Concanavalin-A [ConA] and Griffithsin [GRFT]) during several weeks and we sequenced the genome of the isolated strains. Several potential resistance mutations were identified and characterized by reverse genetics.

## Materials and Methods

### Cell culture

HuH-7-RFP-NLS-IPS were described previously [17] and were obtained by transduction of HuH-7 cells (RCB1366) [18] with Lentivirus pseudoparticles encoding the reporter protein RFP-NLS-IPS [19]. These cells were grown at 37°C, 5% CO_2_ in Dulbecco’s Modified Essential Medium (DMEM, Gibco) supplemented with 10% fetal bovine serum.

### Lectins, antibodies and soluble CD81

ConA and GNA were purchased from Sigma. Purified CV-N was kindly provided by K. Gustafson (National Institutes of Health, National Cancer Institute, Frederick, MD, USA). GRFT was kindly provided by K. E. Palmer (Owensboro Cancer Research Program, Owensboro, Kentucky, USA). The soluble recombinant form of the CD81 large extracellular loop (CD81-LEL) was produced as a glutathione S-transferase fusion protein as described previously [20]. The 3/11 monoclonal antibody (MAb) (anti-E2; kindly provided by J. McKeating, University of Birmingham, United Kingdom) [21] and A4 MAb (anti-E1) [22], were produced *in vitro* by using a MiniPerm apparatus (Heraeus), as recommended by the manufacturer. For neutralization assays, the 3/11 MAb was purified using the Pierce Protein G plus Agarose, as recommended by the manufacturer (Pierce). The C4 MAb (anti-actin) was purchased from Millipore.

### HCV constructs

We used a plasmid encoding JFH1-CS-A4 genome, a modified version of the full-length JFH1 strain (genotype 2a; GenBank access number AB237837; kindly provided by T. Wakita, National Institute of Infectious Diseases, Tokyo, Japan), which contains mutations leading to amino acids changes F172C and P173S at the C-terminus of the Core protein that increase the viral titers [23]. In this construct, the sequence encoding residues ^196^TSSSYMVTNDC at the N-terminal region of E1 has also been modified to reconstitute the A4 epitope (SSGLYHVTNDC) [22], as previously described [24]. Additionally, to characterize the mutations by reverse genetics, we used a plasmid encoding the JFH1-CS-A4-Rluc-DM genome (referred to as wild-type (WT) in this study), which contains a *Renilla* Luciferase reporter gene and two adaptive mutations (R1373Q and C2441S in NS3 and NS5A, respectively), as described previously [17]. These plasmids were used to produce HCV RNA by *in vitro* transcription which were electroporated into HuH-7-RFP-NLS-IPS cells, as previously described [17]. Supernatants of electroporated cells were recovered and filtered through a 0.45-μm-pore-sized membrane, aliquoted and stored until use at -80°C.

### Cell viability assay

HuH-7-RFP-NLS-IPS cells were incubated for 72 h with different concentrations of GNA, CV-N, ConA or GRFT. The viability was measured using the CellTiter-glo® luminescent / Cell viability assay, as recommended by the manufacturer (Promega).

### Selection and isolation of lectin resistant HCV strains

**Fig 1:**
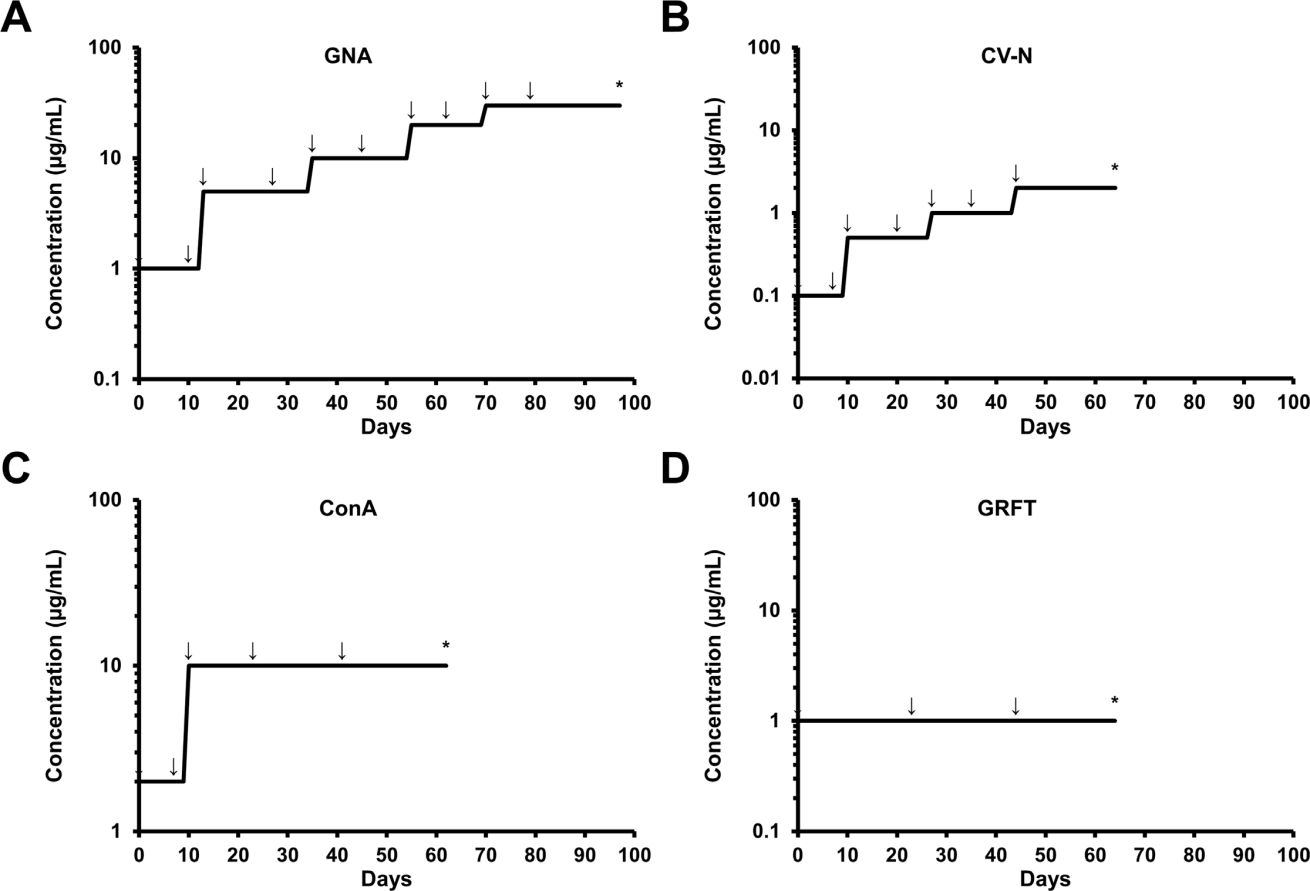
Schedule of drug-escalating selection of GNA, CV-N, ConA and GRFT resistant HCV strains as a function of time. HuH-7-RFP-NLS-IPS cells were infected in the presence of GNA (**A**), CV-N (**B**), ConA (**C**) or GRFT (**D**). Infected cells were subcultured every three to four days in the presence of the lectins. When 100% cells were infected (indicated by arrows), supernatants were recovered and used to infect naive cells in the presence of the lectins. The concentrations of each lectin were increased in a stepwise manner as indicated. Stars indicate the time points when the virus isolates were recovered and sequenced.

HuH-7-RFP-NLS-IPS were infected with a stock of the HCV JFH1-CS-A4 strain (designated i6 stock in [17]) in the presence of the 90% effective concentration of GNA, CV-N, ConA or GRFT (1, 0.1, 2 and 1 μg/mL, respectively). Infected cells were subcultured with administration of the lectins at the same concentration. When 100% cells were infected, the supernatants were recovered and used to infect naive cells in the presence of the lectins. The escalation of lectin concentrations as well as the time points at which viruses were recovered and used to infect naive cells are depicted in Fig 1.

### HCV RNA quantification

Total cellular RNA was extracted using the RNeasy kit (Qiagen). HCV RNA quantification was performed with a real-time RT-PCR assay, as previously described [25].

### Sequencing of viral genomes isolated after selection

HCV RNA in supernatants was extracted using the QIAamp viral RNA mini kit (Qiagen). HCV genome sequences were determined by directly sequencing overlapping purified RT-PCR fragments, with the BigDye Terminator v1.1 kit (Applied Biosystems). The sequence of primers used for RT-PCR and sequencing reactions are available on request.

### Construction and characterization of the mutants

We used the QuickChange II XL Site Directed Mutagenesis Kit (Agilent Technologies) to introduce the potential resistance mutations in the plasmid encoding the JFH1-CS-A4-Rluc-DM genome [17], noted WT in this study. The nucleotide sequences of each mutant were verified. Replication and infectivity were assessed by measuring *Renilla* Luciferase activities using the *Renilla* Luciferase Assay System (Promega) and a Berthold CentroXS3 LB 960 luminometer.

### Inhibition assay

Inhibition assays were performed by preincubating HCVcc with lectins, the 3/11 MAb or CD81-LEL for 1 h at 37°C before they were put in contact with target cells. After 3 h of contact with HCVcc, cells were further incubated for 72 h with complete medium before luciferase activities were measured as indicated by the manufacturer (Promega).

### Statistical analysis

Mann Whitney test was used as indicated, and values of p < 0.05 or p < 0.001 were considered as thresholds for significance.

## Results

### Selection of GNA, CV-N, ConA or GRFT resistant HCV

**Fig 2:**
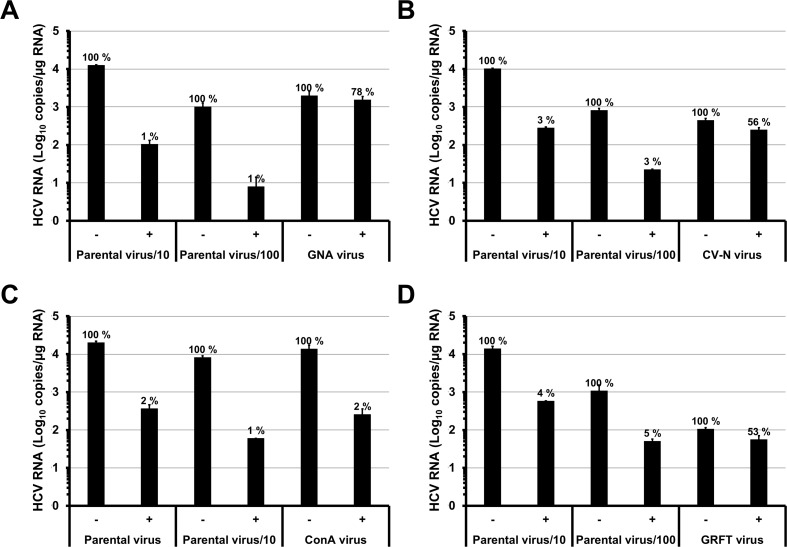
Sensitivity of the strains isolated after selection to inhibition by lectins. HuH-7-RFP-NLS-IPS cells were inoculated with the strains isolated after selection (GNA, CV-N, ConA and GRFT virus) or different dilutions of the parental strain, in the absence (-) or presence (+) of 3 μg/mL of GNA (A), 0.3 μg/mL of CV-N (B), 10 μg/mL of ConA (C) or 1 μg/mL of GRFT (D). After 48 h, cells were lysed and intracellular HCV RNA were quantified by RT-qPCR. Results are expressed as means ± S.D. of duplicates and percentages are indicated.

In order to study viral resistance to CBAs, we cultivated HCV *in vitro* in the presence of increasing concentrations of different high-mannose specific lectins: GNA, CV-N, ConA and GRFT. We took advantage of the HuH-7-RFP-NLS-IPS cell line which enables to monitor HCV infection in living cells [17, 19]. First, we infected HuH-7-RFP-NLS-IPS cells in the presence of the 90% effective concentration of each lectin (1, 0.1, 2 and 1 μg/mL for GNA, CV-N, ConA and GRFT, respectively). Infected cells were then subcultured every three to four days in the presence of the lectin until 100% cells were infected. As soon as all cells were infected, supernatants were recovered and used to infect naive cells in the presence of the lectins. The concentrations of each lectin were increased in a stepwise manner as described in Fig 1. In these conditions, we were able to propagate HCV in the presence of 30 μg/mL of GNA after three months and 2 μg/mL of CV-N after two months. The highest possible ConA concentration was 10 μg/mL due to toxicity of the drug in the cell culture at higher concentrations (S1 Fig). Remarkably, it was difficult to propagate the virus in the presence of GRFT suggesting that adaptation of HCV to this lectin was more challenging. To confirm that we isolated CBA resistant strains, we compared the sensitivity of the isolated strains and the parental strain to lectins. As shown in Fig 2, we observed that the strains isolated in the presence of GNA, CV-N and GRFT were less sensitive to these lectins than the parental strain. In contrast, the strain isolated in the presence of ConA seemed to be as sensitive as the parental strain to ConA.

### Analyses of the envelope genes of HCV strains isolated upon escalating GNA, CV-N, ConA or GRFT exposure

To identify the mutations selected in the E1E2 HCV envelope protein genes during the culture in the presence of each lectin, we sequenced the genomic region encompassing the end of Core to the end of p7. Three mutations were identified after the three month culture with GNA: V284A in E1 and V392D / S449P in E2. Interestingly, the mutation S449P is expected to prevent the addition of the N-glycan on the E2N4 N-glycosylation site at position 448, by introducing a proline residue between the asparagine 448 and the serine 450. The mutation M338V in E2 was found after the culture of HCV in the presence of CV-N, ConA and GRFT. Interestingly, we also identified the mutation L755M in p7 in the three viruses isolated after culture with these lectins. Three other mutations, L612M, N417S and A400D, were also detected in the viruses selected with CV-N, ConA and GRFT, respectively. The N417S mutation has previously been shown to lead to a shift of the E2N1 N-glycan from N417 to N415 [26]. Importantly, when the virus was passaged in absence of lectin, we only detected the mutation I599V in E2. These mutations are summarized in bold characters in Table 1. Using the European Hepatitis C Virus database (euHCVdb) [27], we observed that mutations V284A, N417S, S449P and L755M occurred on residues which are highly conserved not only in genotype 2a HCV sequences but also in sequences of other genotypes (Table 2). In these sequences, only three synonymous mutations were identified (one in the end of Core, and two in p7, after GNA exposure). Altogether, these results suggest that a positive selection occurred on the HCV envelope protein genes in the presence of lectins. This was also supported by the cumulative behavior of the dS/dN ratio, obtained using the Synonymous / Non-synonymous Analysis Program (http://www.hiv.lanl.gov/content/sequence/SNAP/SNAP.html) [28] and comparing the four sequences obtained after lectin exposure and the control sequence obtained after culture without lectin (S2 Fig).

**Table 1:** Features of the lectin resistant HCV selections.

Lectin	Dose (μg/mL)		Identified mutations (protein)
	Start	End	
GNA	1	30	I30T^¶^ (Core) ; V284A (E1) ; V392D (E2) ; S449P* (E2) ; N2081H (NS5A) ; P2271S^¶^ (NS5A) ; I2340T (NS5A) ; C2441S^¶^ (NS5A) ; V2564A (NS5B) ; A2592V (NS5B)
CV-N	0.1	2	**M338V (E1) ; L612M (E2) ; L755M (p7)** ; F1926I (NS4B) ; I2270L^¶^ (NS5A) ; E2410G (NS5A)
ConA	2	10	T11A (Core) ; **M338V (E1) ; N417S**^**#**^** (E2) ; L755M (p7)** ; F1926I (NS4B) ; D2227V^¶^ (NS5A) ; K2542Q (NS5B)
GRFT	1	1	K12N (Core) ; **M338V (E1) ; A400D (E2) ; L755M (p7)** ; F1926I (NS4B) ; D2254G^¶^ (NS5A) ; M2275V (NS5A) ; Y2718H (NS5B)
Mock	-	-	I599V^+^ (E2) ; R1373Q^+^^¶^ (NS3) ; M1611T^+^^¶^ (NS3) ; S2364P^+^ (NS5A) ; C2441S^+^^¶^ (NS5A) ; R2523K^+^ (NS5B)

*: This mutation prevents the modification of E2N4 N-glycosylation site on asparagine 448.

#: This mutation leads to a shift of the E2N1 N-glycosylation site and improves HCV entry.

+: These mutations have been characterized in a previous study [17].

¶: Similar mutations have already been identified in JFH1 cell culture adaptation in absence of lectin [17, 29–36].

**Table 2:** Conservation of the mutated amino acids.

Mutation (protein)	% conservation (sequence number)	
	all genotypes	genotype 2a
V284 (E1)	86.8% (2556/2945)	95.2% (20/21)
M338 (E1)	4.1% (122/2945)	95.2% (20/21)
V392 (E2)	11.2% (232/2073)	22.2% (4/18)
A400 (E2)	28.9% (599/2073)	55.6% (10/18)
N417 (E2)	97.6% (2024/2073)	100% (18/18)
S449* (E2)	84.0% (1741/2073)	100% (18/18)
I599 (E2)	1.7% (35/2073)	72.2% (13/18)
L612 (E2)	56.1% (1162/2073)	88.9% (16/18)
L755 (p7)	99.5% (1449/1456)	100% (17/17)

*: The mutation S449P prevents the modification of the E2N4 N-glycosylation site which is conserved in 97.6% of sequences of all genotypes and 100% of genotype 2a sequences.

### Effect of the E1E2p7 mutations selected upon lectin exposure on replication and infectious virus production

**Fig 3:**
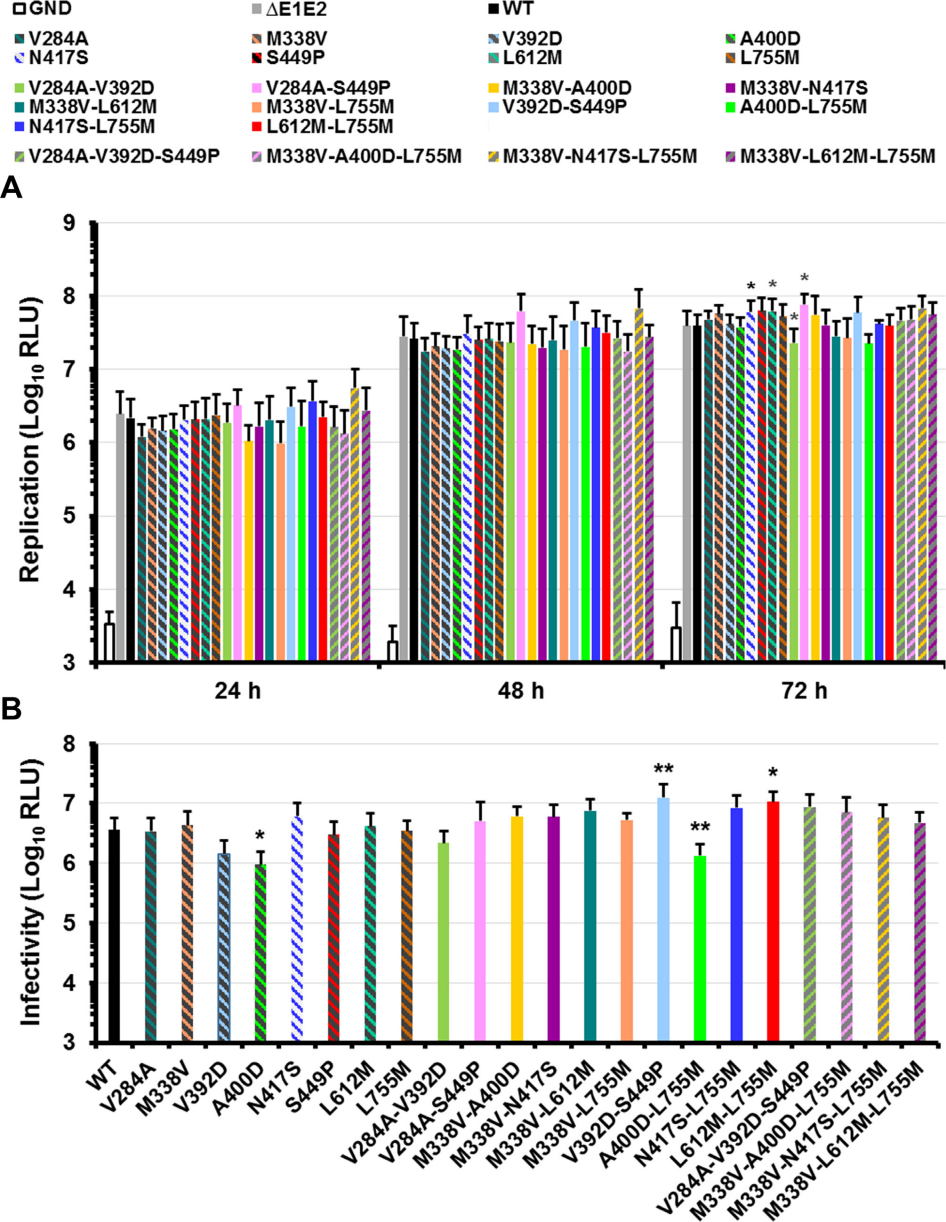
Effect of the E1E2p7 mutations on viral genome replication and infectious virus production. (**A**) HuH-7-RFP-NLS-IPS cells were transfected with WT or mutated HCV genomes. An assembly-deficient virus (ΔE1E2) and a replication-defective virus (GND) were used as controls. Replication was assessed at 4, 24, 48 and 72 h by measuring *Renilla* Luciferase activities in transfected cells. Results are expressed as relative light units (RLU) normalized at 4 h and are reported as the means ± standard deviation (S.D.) of at least three independent experiments. (**B**) The supernatants of transfected cells were recovered at 48 h and incubated for 4 h with naive HuH-7-RFP-NLS-IPS cells. Luciferase assays were performed on infected cells at 72 h post-infection. Results are normalized by the replication at 48 h, expressed as RLU and are reported as the means ± S.D. of at least three independent experiments. (*: p < 0.05; **: p < 0.001). Luciferase activities obtained with ΔE1E2 supernatants were around 10^3^ RLU.

**Fig 4:**
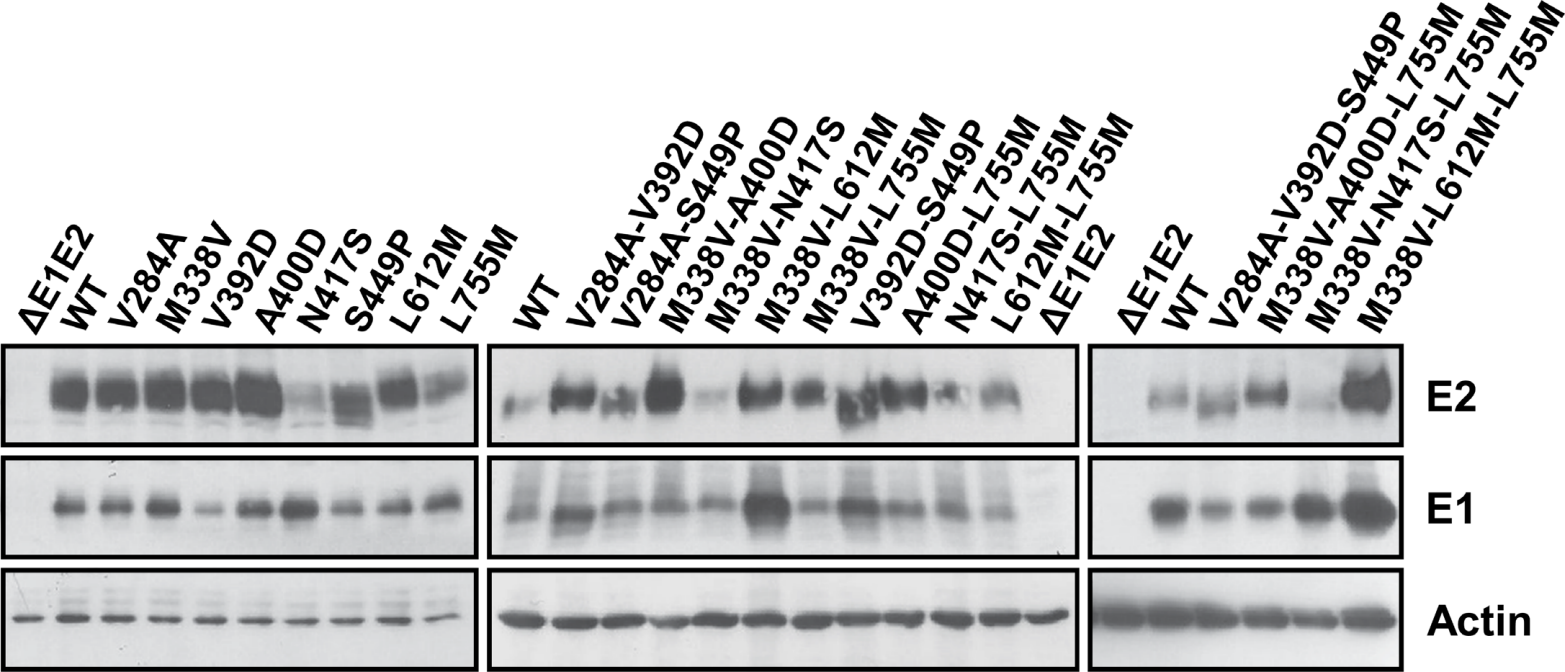
Effect of the E1E2p7 mutations on E1E2 glycoprotein expression. HuH-7-RFP-NLS-IPS cells were transfected with WT or mutated HCV genomes. An assembly-deficient virus (ΔE1E2) was used as control. Expression of E1E2 viral glycoproteins and actin was analyzed in cell lysates 72h post-electroporation by western blotting with specific MAbs (A4 [anti-E1], 3/11 [anti-E2] and C4 [anti-actin]).

To study the effect of the E1E2 mutations on the virus fitness, we introduced them into a modified JFH1 genome expressing a *Renilla* Luciferase reporter gene by reverse genetics [17]. Since the mutation L755M in p7 was fixed in three independent lectin exposures, we also decided to characterize it. Mutations were introduced alone or by combination of 2 or 3. A replication- defective virus carrying a mutation in the NS5B GDD motif (GND) and a variant genome carrying a large in-frame deletion in the E1E2 coding region known to inactivate viral particle release (ΔE1E2) were used as negative controls for replication and assembly, respectively [16]. First, we assessed the impact of the mutations on HCV RNA replication. As shown in Fig 3A, some individual mutations or combinations of two mutations led to slight variations of the replication measured as Luciferase activities 24, 48 and 72 h after electroporation. An increased replication was observed at 72 h for the N417S, L612M and V284A-S449P mutants (1.6-, 1.6- and 2.0-fold WT respectively, p < 0.05). In contrast, the mutant V284A-V392D showed a slight decrease of replication (0.6-fold WT, p < 0.05). However, the combinations of three mutations did not have any significant effect on replication as compared to WT (1.2-, 1.2-, 1.7- and 1.4-fold WT at 72 h for V284A-V392D-S449P / M338V-A400D-L755M / M338V-N417S-L755M / M338V-L612M-L755M, respectively). Using western blotting on electroporated cells, we observed that E2 proteins containing the S449P mutation migrated slightly faster than the WT E2 which confirmed that this mutation leads to an impediment of the asparagine 448 N-glycosylation (Fig 4). In addition, the detection of E2 for the mutants containing the N417S substitution was often weaker than the WT E2 because this residue is involved in the epitope of the 3/11 MAb which was used for the western blot. To further characterize our mutants, infectious virus production was measured at 48 h post-electroporation for each mutant and compared to that of the WT. Supernatant of cells electroporated with the ΔE1E2 mutants was used as negative control to determine the background level of Luciferase activity. As shown in Fig 3B, the production of infectious virus for A400D and A400D-L755M mutants was significantly decreased as compared to that of the WT (3.0- and 2.5-fold WT, p < 0.05 and p < 0.001, respectively). On the contrary, results suggest that other combinations of two mutations positively modulate infectivity. The production of infectious viral particles was markedly improved for the V392D-S449P and L612M-L755M mutants (3.5- and 3.0-fold WT, p < 0.001 and p < 0.05, respectively). However, the different combinations of three mutations that were found at the end of each selection did not have any significant effect on infectious viral particle production.

### Effect of the E1E2p7 mutations selected upon lectin exposure on sensitivity to lectin inhibition

**Fig 5:**
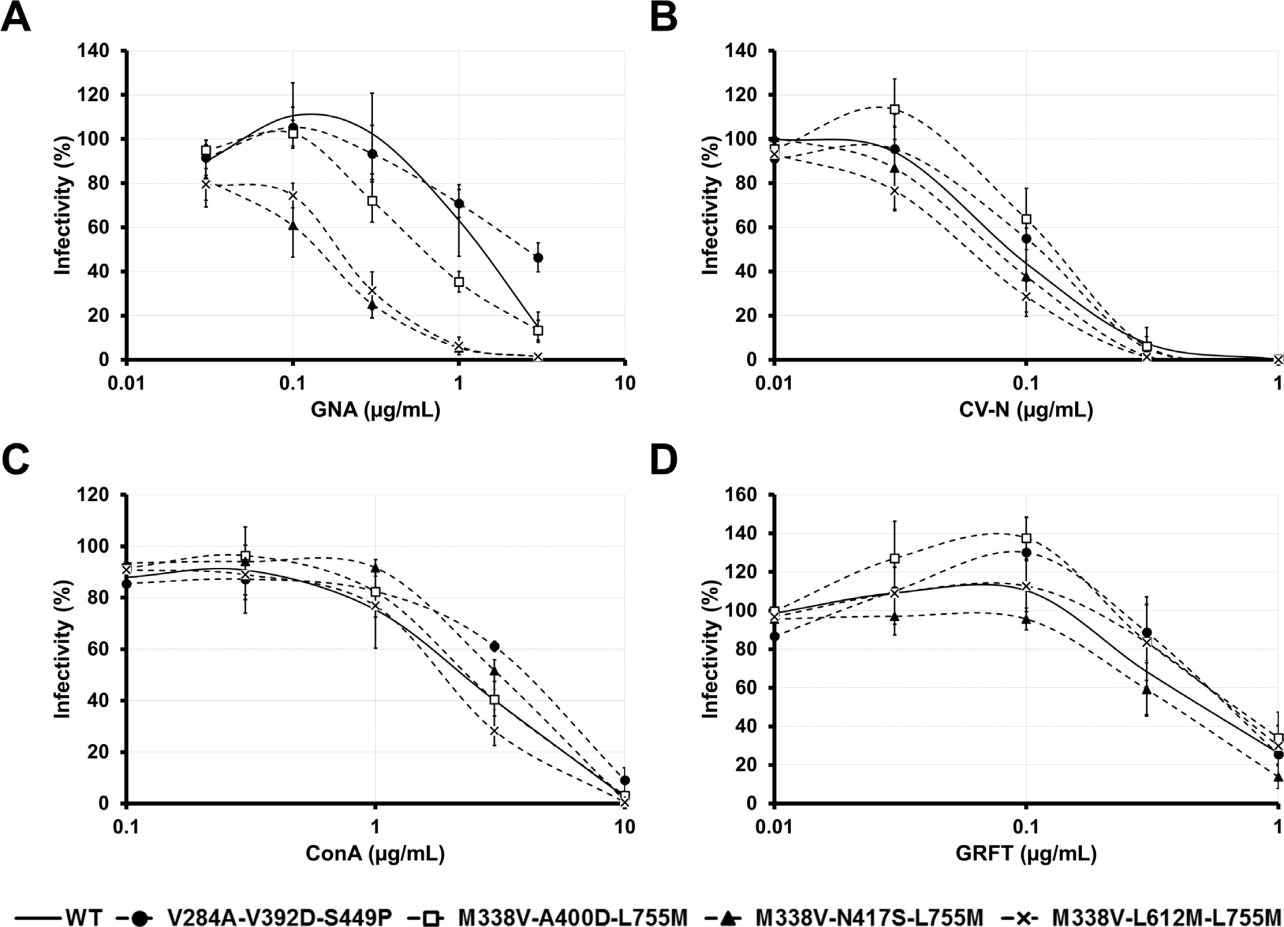
Effect of the E1E2p7 mutations on sensitivity to inhibition by GNA, CV-N, ConA and GRFT. Inhibition assays were performed by incubating WT or mutant HCVcc with various concentrations of GNA (**A**), CV-N (**B**), ConA (**C**) or GRFT (**D**). After a 1 h incubation at 37°C, mixes were put into contact with target cells for 4 h. Luciferase assays were performed on infected cells at 72 h post-infection. Results are expressed as percentages of infectivity compared to infection in absence of inhibitory protein and are reported as the means ± S.D. of at least three independent experiments.

In order to determine whether the mutations selected upon lectin exposure confer resistance to the lectins, we performed dose response curves and compared the ability of the lectins to inhibit WT HCVcc and the mutants containing the combinations of three mutations. As shown in Fig 5, we observed that the combinations of three mutations V284A-V392D-S449P, M338V-L612M-L755M, M338V-N417S-L755M and M338V-A400D-L755M did not confer any resistance to the lectin they have been selected with (GNA, CV-N, ConA and GRFT, respectively). We also analyzed the effect of simple mutation and combination of two mutations on the sensitivity to inhibition by lectins. In the same way, we did not evidence any resistance conferred by the mutations selected upon GNA, CV-N or ConA exposure, alone or in combination of two (S3 Fig). The results obtained with A400D, M338V-A400D and A400D-L755M mutants were surprising since at low concentrations of GRFT (between 0.03 μg/mL and 0.3 μg/mL), the infectivity was increased (S3 Fig). This could suggest that the A400D mutation confers a susceptibility to a beneficial conformational change induced by low concentration of GRFT.

We also tested the sensitivity of the four triple mutants to inhibition by other lectins and observed that they were equally sensitive to CV-N, ConA and GRFT (Figs 5B and 4C and 4D). In contrast, we noticed that mutants M338V-N417S-L755M, M338V-L612M-L755M and M338V-A400D-L755M were more sensitive to GNA inhibition (Fig 5A). These combinations of mutations led to a 9.8-, 7.3- and 2.3-fold reduction in the effective concentration required for half-maximal inhibition as compared to WT, respectively.

### Effect of the E1E2p7 mutations selected upon lectin exposure on sensitivity to 3/11 MAb and soluble CD81 inhibition

**Fig 6:**
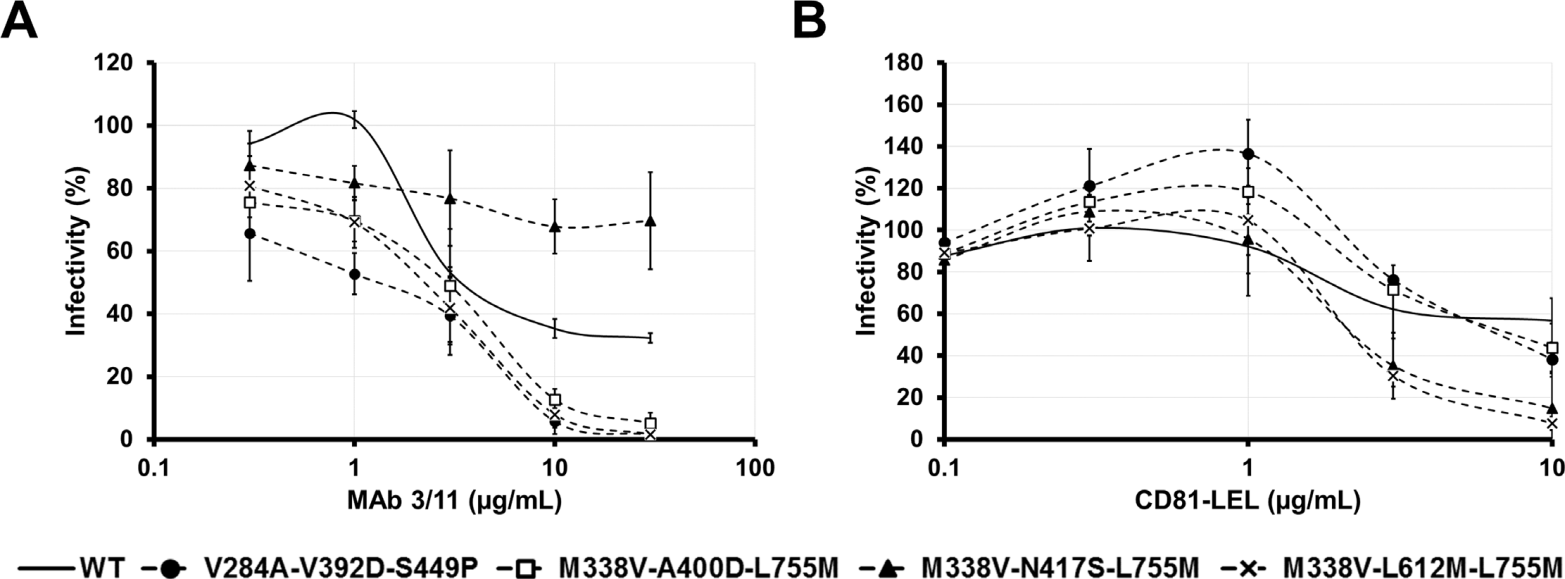
Effect of the E1E2p7 mutations on sensitivity to inhibition by MAb 3/11 and CD81-LEL. Inhibition assays were performed by incubating WT or mutant HCVcc with various concentrations of MAb 3/11 (**A**) or CD81-LEL (**B**). After a 1 h incubation at 37°C, mixes were put into contact with target cells for 4 h. Luciferase assays were performed on infected cells at 72 h post-infection. Results are expressed as percentages of infectivity compared to infection in absence of inhibitory protein and are reported as the means ± S.D. of at least three independent experiments.

Since some of the mutations identified upon lectin exposure affected the N-glycosylation of E2 envelope protein which is known to protect the CD81 binding site from neutralizing antibodies, we also investigated the mutant sensitivity to the 3/11 neutralizing MAb and to a soluble form of the CD81 large extracellular loop (CD81-LEL). As shown in Fig 6A, we observed that V284A-V392D-S449P, M338V-L612M-L755M and M338V-A400D-L755M mutants were more sensitive to 3/11 neutralization than WT (more than 95% inhibition at 30 μg/mL compared to about 70% for WT) whereas M338V-N417S-L755M mutant was resistant (only 30% inhibition at 30 μg/mL). In addition, we noticed that M338V-L612M-L755M and M338V-N417S-L755M mutants were slightly more sensitive to CD81-LEL inhibition than WT (more than 85% inhibition at 10 μg/mL compared to about 50% for WT; Fig 6B).

### Analyses of the Core and the non-structural protein genes of HCV strains isolated upon escalating GNA, CV-N, ConA or GRFT exposure

To explain how the virus succeeded to propagate in the presence of some lectins, we hypothesized that other mutations which improve the virus fitness or modify the property of the viral particles could have been fixed upon lectin exposure. For this reason, we sequenced the regions encoding the Core and the non-structural proteins of the strains isolated upon lectin exposure. This enabled us to identify several additional mutations (7, 3, 4 and 5 upon GNA, CV-N, ConA and GRFT exposure, respectively). These mutations are presented in non-bold characters in Table 1. Interestingly, similar mutations at some of these positions had already been identified by other groups during JFH1 cell culture adaptation (I30S [29], P2271L [30], C2441S [17, 31–33], I2270T [33–35], D2227V [36], D2254G [34]) and several of these mutations have been shown to be beneficial for the virus, in particular for the assembly process. Furthermore, the cumulative behavior of the dS/dN ratio (S2 Fig) also evidenced that the region encoding the end of NS5A, which is essential for cell culture adaptation, was under positive selection. Altogether, our results suggest that HCV resistance to CBAs is not directly conferred by mutations in the E1E2 envelope protein genes but could occur through an indirect mechanism involving mutations in other viral proteins. Further investigations are needed to completely elucidate the mechanisms of HCV resistance to CBAs.

## Discussion

In this study, we aimed at evaluating the resistance of HCV to CBAs *in vitro*. After more than eight weeks of HCV culture in the presence of increasing concentrations of different lectins, we identified several mutations in the genomes of the isolated strains, and evidenced a positive selection on the E1E2 coding region, as a consequence of the V284A, M338V, V392D, A400D, N417S, S449P and L612M non-synonymous mutations. Other mutations were also detected in the regions encoding the Core and the non-structural proteins of the isolated strains, with a second region under positive selection corresponding to the end of NS5A, a region known to be frequently mutated during cell culture adaptation. The characterization of the E1E2 mutations, alone or in combination, evidenced that none of these mutations confer resistance to CBAs. Importantly, when similar experiments were performed in the presence of neutralizing MAbs, escape mutations were observed as early as 5 days post treatment [26, 37–39]. Several explanations can be put forward to explain the difference between HCV resistance to MAbs and CBAs. Firstly, it is important to note that E1 and E2 bring many conserved N-glycans, thus many CBA targets, which have been shown to play an important role in the different steps of the HCV life cycle. Moreover, it has to be noticed that in contrast to neutralizing antibodies, CBAs are able to inhibit cell free as well as cell to cell transmission [14]. Finally, it has also been proposed that some lectins could act through both viral and cellular glycan interactions [13].

In case of Human Immunodeficiency Virus (HIV), it has been proposed that a CBA-based therapeutic strategy could not only directly inhibit viruses, but also induce partial loss of the glycan shield and make viruses more vulnerable to attack by the immune system [40, 41]. Interestingly, we observed that even if no resistance mutation could be evidenced in the E1E2 HCV envelope glycoproteins, the positive selection which occurred led to a modification of the HCV glycan shield. Indeed, among the identified mutations, the S449P amino acid change prevents the N-glycosylation of the asparagine 448 residue corresponding to the E2N4 glycosylation site which is important for HCV particle assembly and infectivity as well as protection against neutralization. Using the 3/11 neutralizing MAb, we confirmed that this mutation renders the viral particle more sensitive to neutralization. We also identified the mutation N417S which has previously been thoroughly characterized. This mutation (or the related mutation N417T) has been described as an adaptive mutation enhancing the entry process *in vitro* [42–46]. In our study, this mutation was responsible for a 1.7-fold increase of the infectivity but this was not significant. This mutation is also known to lead to a shift of the E2N1 N-glycan from N417 to N415, which prevents the recognition of N415 residue by several neutralizing antibodies, as observed here with the 3/11 neutralizing MAb [26, 37, 38, 47, 48]. Interestingly, dose response curves using single, double and triple mutants suggest that this mutation was also responsible for an increased sensitivity to GNA (Fig 5A and data not shown). Since GNA binds to mannose termini and lactosamine structures that are present in high-mannose-, hybrid- and complex-type N-glycans, the increased sensitivity may not be caused by a difference of N-glycan maturation. Instead, a better accessibility of the E2N1 N-glycan to the GNA could explain this feature. Interestingly, our results reveal that the mutation L612M, which may be critical for the overall E2 architecture [49], also increases the sensitivity to GNA (Fig 5A and data not shown). However, caution must be taken because such mutations which do not confer lectin resistance but increase the sensitivity to neutralizing antibodies may not be fixed *in vivo*, in the presence of the humoral immunity. Thus, in contrast to HIV [40, 41], it seems unlikely that development of HCV resistance against CBAs would result in an enhancement of neutralization by the immune system.

With the recent approval of several direct-acting antivirals inhibiting HCV NS3/4A, NS5A or NS5B proteins, CBAs and more generally viral entry inhibitors are unlikely to find an indication for HCV treatment [50]. At best, it may be used for the prevention of graft reinfection in HCV-infected liver transplant patients or possibly for the treatment of HCV-HIV coinfected patients where drug interactions with antiretrovirals are frequently observed. However, an important feature of CBA-based therapeutic strategy is the broad antiviral spectrum. In particular, CBAs have been shown to efficiently inhibit several emergent viruses such as Dengue Virus [51], Ebola Virus [52, 53] or Severe Acute Respiratory Syndrome Coronavirus [54, 55]. To our knowledge, viral resistance to CBAs had only been evaluated using HIV strains, which are characterized by an evolving glycan shield. Thus, our results bring new insights into the development of CBA-based antiviral strategy since they suggest that HCV resistance to CBAs is not directly conferred by mutations in the E1E2 envelope protein genes but could occur through an indirect mechanism involving mutations in other viral proteins. Further investigations are needed to completely elucidate the underlying mechanisms.

## Supporting Information

S1 Fig GNA, CV-N, ConA and GRFT toxicity on HuH-7-RFP-NLS-IPS cells. HuH-7-RFP-NLS-IPS cell viability was evaluated 3 days after incubation with 0, 1, 3, 10, 30 or 100 μg/mL of GNA, CV-N, ConA or GRFT. The results are expressed as percentages of viability compared to the viability in absence of lectin and are reported as the means ± S.D. of three independent experiments.
(TIF)

S2 Fig Cumulative behavior of the dS/dN ratio. The cumulative behavior of the dS/dN ratio obtained using the Synonymous / Non-synonymous Analysis Program (http://www.hiv.lanl.gov/content/sequence/SNAP/SNAP.html) and comparing the four sequences obtained after lectin exposure and the control sequence obtained after culture without lectin is shown. The regions encoding the different HCV proteins are depicted on the top.
(TIF)

S3 Fig Effect of the simple and double mutations in E1E2p7 on sensitivity to inhibition by GNA, CV-N, ConA and GRFT. Inhibition assays were performed by incubating WT or simple and double mutant HCVcc with various concentrations of GNA (**A**), CV-N (**B**), ConA (**C**) or GRFT (**D**). After a 1 h incubation at 37°C, mixes were put into contact with target cells for 4 h. Luciferase assays were performed on infected cells at 72 h post-infection. Results are expressed as percentages of infectivity compared to infection in absence of inhibitory protein and are reported as the means ± S.D. of at least three independent experiments.
(TIF)
