# Supplementary figures and images for: Hepatitis C Virus Resistance to Carbohydrate-Binding Agents
Source: PLoS One. 2016 Feb 12;11(2):e0149064. doi: 10.1371/journal.pone.0149064 (PMC4752358; doi:10.1371/journal.pone.0149064)

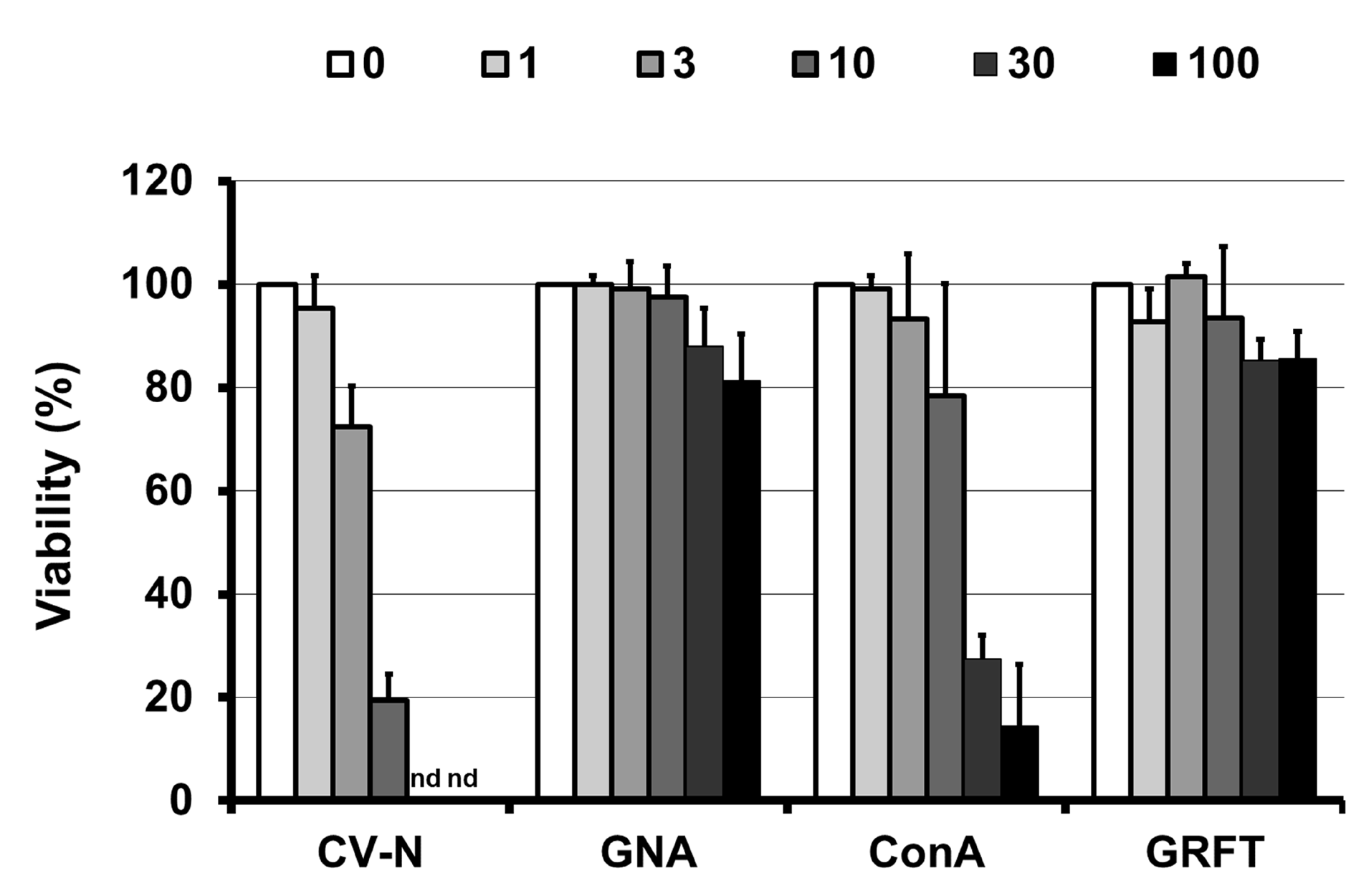

Supplement: S1 Fig — HuH-7-RFP-NLS-IPS cell viability was evaluated 3 days after incubation with 0, 1, 3, 10, 30 or 100 μg/mL of GNA, CV-N, ConA or GRFT. The results are expressed as percentages of viability compared to the viability in absence of lectin and are reported as the means ± S.D. of three independent experiments. (TIF) [file pone.0149064.s001.tif]

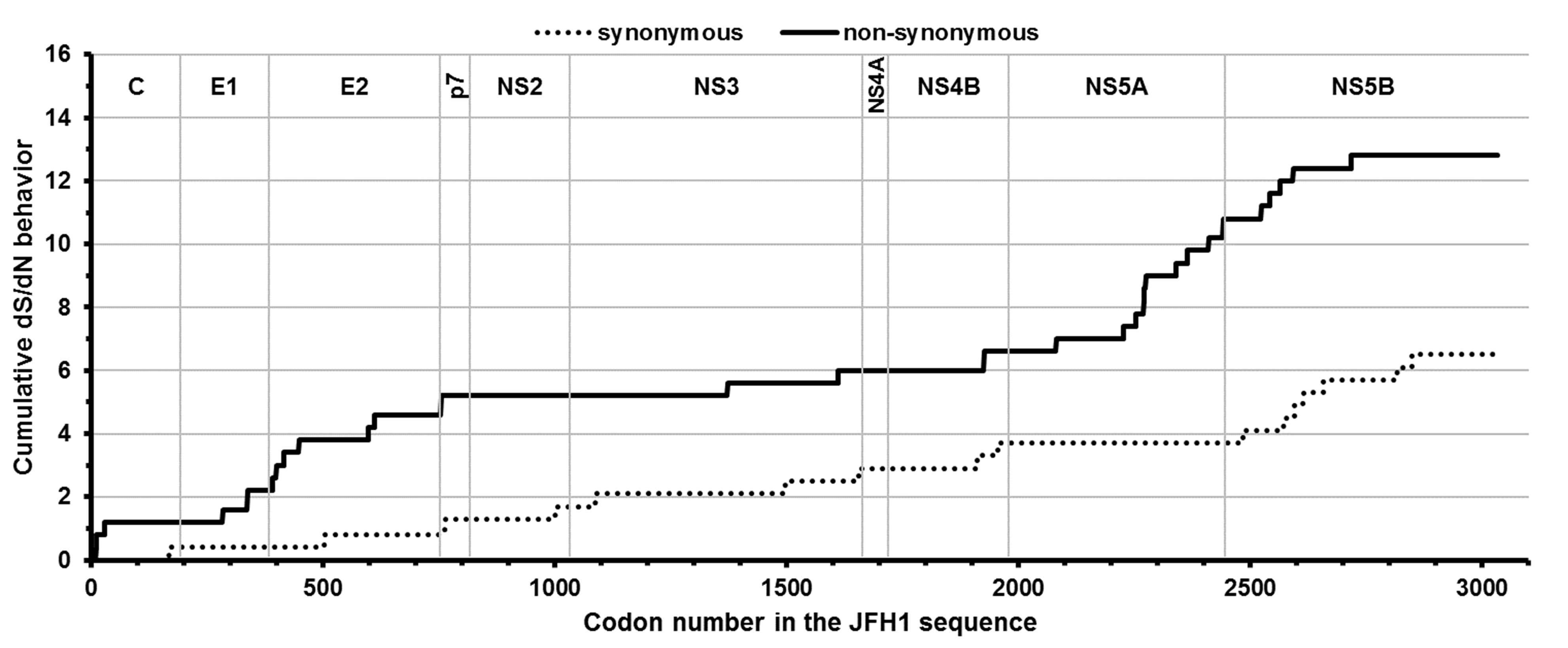

Supplement: S2 Fig — The cumulative behavior of the dS/dN ratio obtained using the Synonymous / Non-synonymous Analysis Program (http://www.hiv.lanl.gov/content/sequence/SNAP/SNAP.html) and comparing the four sequences obtained after lectin exposure and the control sequence obtained after culture without lectin is shown. The regions encoding the different HCV proteins are depicted on the top. (TIF) [file pone.0149064.s002.tif]

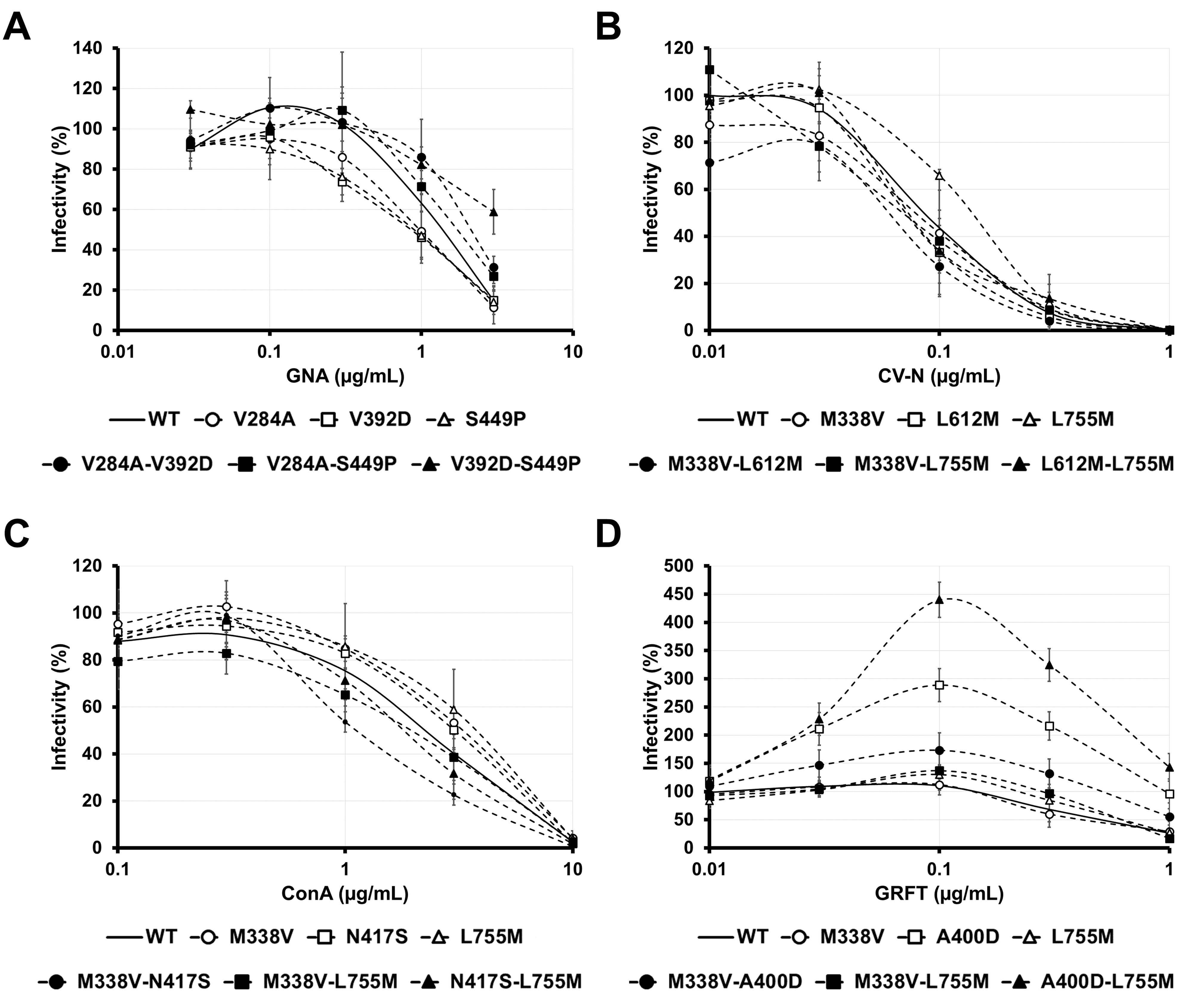

Supplement: S3 Fig — Inhibition assays were performed by incubating WT or simple and double mutant HCVcc with various concentrations of GNA (A), CV-N (B), ConA (C) or GRFT (D). After a 1 h incubation at 37°C, mixes were put into contact with target cells for 4 h. Luciferase assays were performed on infected cells at 72 h post-infection. Results are expressed as percentages of infectivity compared to infection in absence of inhibitory protein and are reported as the means ± S.D. of at least three independent experiments. (TIF) [file pone.0149064.s003.tif]
